# Altered Entrainment to the Day/Night Cycle Attenuates the Daily Rise in Circulating Corticosterone in the Mouse

**DOI:** 10.1371/journal.pone.0111944

**Published:** 2014-11-03

**Authors:** Patricia J. Sollars, Michael J. Weiser, Andrea E. Kudwa, Jayne R. Bramley, Malcolm D. Ogilvie, Robert L. Spencer, Robert J. Handa, Gary E. Pickard

**Affiliations:** 1 Neuroscience Division, Department of Biomedical Sciences, Colorado State University, Fort Collins, Colorado, 80523, United States of America; 2 School of Veterinary Medicine and Biomedical Sciences, University of Nebraska, Lincoln, Nebraska, 68583, United States of America; 3 Department of Psychology and Neuroscience, University of Colorado, Boulder, Colorado, 80309, United States of America; 4 Department of Basic Medical Sciences, University of Arizona College of Medicine, Phoenix, Arizona, 85004, United States of America; University of Lübeck, Germany

## Abstract

The suprachiasmatic nucleus (SCN) is a circadian oscillator entrained to the day/night cycle via input from the retina. Serotonin (5-HT) afferents to the SCN modulate retinal signals via activation of 5-HT_1B_ receptors, decreasing responsiveness to light. Consequently, 5-HT_1B_ receptor knockout (KO) mice entrain to the day/night cycle with delayed activity onsets. Since circulating corticosterone levels exhibit a robust daily rhythm peaking around activity onset, we asked whether delayed entrainment of activity onsets affects rhythmic corticosterone secretion. Wheel-running activity and plasma corticosterone were monitored in mice housed under several different lighting regimens. Both duration of the light∶dark cycle (T cycle) and the duration of light within that cycle was altered. 5-HT_1B_ KO mice that entrained to a 9.5L:13.5D (short day in a T = 23 h) cycle with activity onsets delayed more than 4 h after light offset exhibited a corticosterone rhythm in phase with activity rhythms but reduced 50% in amplitude compared to animals that initiated daily activity <4 h after light offset. Wild type mice in 8L:14D (short day in a T = 22 h) conditions with highly delayed activity onsets also exhibited a 50% reduction in peak plasma corticosterone levels. Exogenous adrenocorticotropin (ACTH) stimulation in animals exhibiting highly delayed entrainment suggested that the endogenous rhythm of adrenal responsiveness to ACTH remained aligned with SCN-driven behavioral activity. Circadian clock gene expression in the adrenal cortex of these same animals suggested that the adrenal circadian clock was also aligned with SCN-driven behavior. Under T cycles <24 h, altered circadian entrainment to short day (winter-like) conditions, manifest as long delays in activity onset after light offset, severely reduces the amplitude of the diurnal rhythm of plasma corticosterone. Such a pronounced reduction in the glucocorticoid rhythm may alter rhythmic gene expression in the central nervous system and in peripheral organs contributing to an array of potential pathophysiologies.

## Introduction

The suprachiasmatic nucleus (SCN) is a compact group of small neurons located in the anterior hypothalamus wherein both neural activity and the expression of a set of core ‘clock’ genes oscillate in a circadian fashion with a period of approximately 24 h in the absence of environmental cues [1], [2]. The circadian oscillations of the SCN neuronal network regulate the temporal aspect of mammalian behavior, physiology and metabolism. The SCN receives direct information regarding the level of ambient illumination via the retinohypothalamic tract (RHT) [3]. These retinal signals entrain the molecular and neurophysiologic circadian oscillations of the SCN to the day/night cycle providing stable phasing of cyclic behavior and endocrine rhythms in relation to the environment. It is the appropriate phasing of the SCN oscillator with the environment that, in effect, enables recognition of local time. Thus, the SCN circadian oscillator is said to function as a biological clock [2].

Photic entrainment of the endogenous SCN circadian oscillation is accomplished by a daily resetting of the SCN clock such that the daily light-induced phase shift is equal in magnitude to the difference between the free-running period of the endogenous SCN oscillation and the period of the day/night cycle (i.e., typically 24 h but importantly not restricted to this period). Light exposure early in the subjective night delays the SCN oscillation and light experienced around dusk (or light offset under laboratory conditions) is the prominent photic cue used for entrainment when the period of the endogenous SCN circadian oscillation is less than the environmental cycle, as is typical for most strains of laboratory mice. Light exposure late in the subjective night results in phase advances of the SCN and thus light around dawn (or light onset in the laboratory) is the salient photic cue used to reset the SCN clock of organisms with a period longer than the environmental cycle, such as most humans on earth. The SCN clock of both nocturnal and diurnal animals is relatively insensitive to the phase shifting effects of light during most of the subjective day [2], [3].

Photic signals responsible for entraining the SCN to the day/night cycle arise primarily from intrinsically photosensitive retinal ganglion cells (ipRGCs) that express the novel photopigment melanopsin [4]–[6]. In the mouse the RHT is composed entirely of axons from ipRGCs [7], [8] and more than one type of ipRGC contributes to the RHT [7]–[10]. RHT input to the SCN is modulated by serotonergic afferents from the median raphe nucleus [11]. Activation of presynaptic 5-HT_1B_ receptors located on ipRGC terminals [12]–[13] inhibits light-induced behavioral phase shifts, light-induced expression of the immediate-early gene *c-fos* in the SCN, light-induced suppression of pineal melatonin synthesis [14], [15] and reduces the amplitude of glutamatergic excitatory postsynaptic currents in the SCN evoked by selectively stimulating the RHT [12], [16].

The responses of 5-HT_1B_ receptor knockout (5-HT_1B_ KO) mice to light are diminished [17], [18]. This seemingly paradoxical reduction in light sensitivity after the loss of inhibitory presynaptic 5-HT_1B_ receptors on RHT terminals is apparently due to the concomitant disinhibition of GABA release in the SCN resulting from the loss of inhibitory presynaptic 5-HT_1B_ receptors on GABA terminals [13], [19]. The attenuated response to light of the SCN of 5-HT_1B_ KO mice is evident when these animals are maintained in light∶dark (L∶D) cycles of less than 24 h in duration. Such lighting conditions mandate relatively large daily light-induced phase advances of the SCN clock to achieve stable entrainment [17]. 5-HT_1B_ KO mice entrain to 22 h and 23 h L∶D cycles with delayed onsets of locomotor activity that effectively result in a greater portion of their late subjective night being coincident with light around dawn (i.e., light onset), the phase of the circadian cycle when light generates advance phase shifts [17]. Thus, the 5-HT_1B_ KO mouse maintained in 23 h L∶D cycles is a useful model to study the potential detrimental effects of altered entrainment to the day/night cycle.

Adrenal glucocorticoid secretion (cortisol in humans and corticosterone in mice) is one of many hormones that exhibit circadian variation; circulating glucocorticoid levels typically peak shortly before activity onset in both nocturnal rodents and diurnal humans [20]. Corticosterone (CORT) secretion from the adrenal cortex is dependent on adrenocorticotropic hormone (ACTH) release from the anterior pituitary gland and ACTH secretion parallels that of CORT, although the daily rhythm in ACTH is of much lower amplitude [21]. However, early studies indicated that rhythmic ACTH was not required for rhythmic CORT secretion as the rhythm persisted in hypophysectomized animals with constant ACTH replacement [22]. It is now well documented that the autonomic innervation of the adrenal gland plays an important role in regulating the diurnal rhythm of CORT secretion [23]–[27].

It has also become apparent that both the rhythmicity of CORT secretion and its amplitude convey important physiologic information. Glucocorticoids are potent transcriptional regulators and rhythmic gene expression in several regions of the central nervous system is critically dependent on the daily rhythm of CORT secretion [28]–[32]. The daily rhythm in CORT also plays a key role in synchronizing subordinate circadian oscillators in peripheral tissues by activating core clock genes [33], [34]. Moreover, flattening of the daily CORT rhythm alters monoaminergic neurotransmission, metabolism, and is correlated with survival in cancer patients [35]–[37]. A flattening in the daily cortisol rhythm due to increased basal levels is also common for patients suffering from major depression [38]–[41]. Thus alterations in the daily rhythm of plasma CORT exert a wide range of effects on metabolism and central nervous system function and mood.

Since rhythmic cortisol secretion is believed to be regulated by the SCN clock, pathological conditions associated with altered circadian rhythms may simultaneously affect rhythmic cortisol secretion. Recurrent winter depression or seasonal affective disorder (SAD) is generally thought to be associated with phase-delayed biological rhythms [42]–[44] and therefore the cortisol rhythm may be similarly affected in SAD patients. Although changes in cortisol secretion are well documented in non-seasonal unipolar depression, only a few studies of the hypothalamic-pituitary-adrenal (HPA) axis have been conducted in SAD patients and this type of atypical depression has more frequently been associated with abnormally low HPA activity [45], [46]. Avery and colleagues reported both a phase delay and a reduction in amplitude in the plasma cortisol rhythm in SAD patients [46] although others have not observed these changes in SAD patients [47], [48]. The extent to which the daily rhythm of plasma CORT is dependent on the phase relationship of the SCN circadian oscillator to the day/night cycle in animals is unexplored. Since the 5-HT_1B_ KO mouse with an attenuated response to light manifests an abnormal delay in the phase relationship of the circadian system to light offset [17], we sought to determine if the daily rhythm of CORT is altered in 5-HT_1B_ KO mice with abnormal entrainment to the day/night cycle. The results indicate that when the circadian system is phase delayed by more than approximately 4 hours, the diurnal rhythm of plasma CORT is significantly attenuated.

## Results

### Experiment 1: CORT rhythms in 5-HT_1B_ KO and WT mice under 9.5L:13.5D conditions

**Figure 1:**
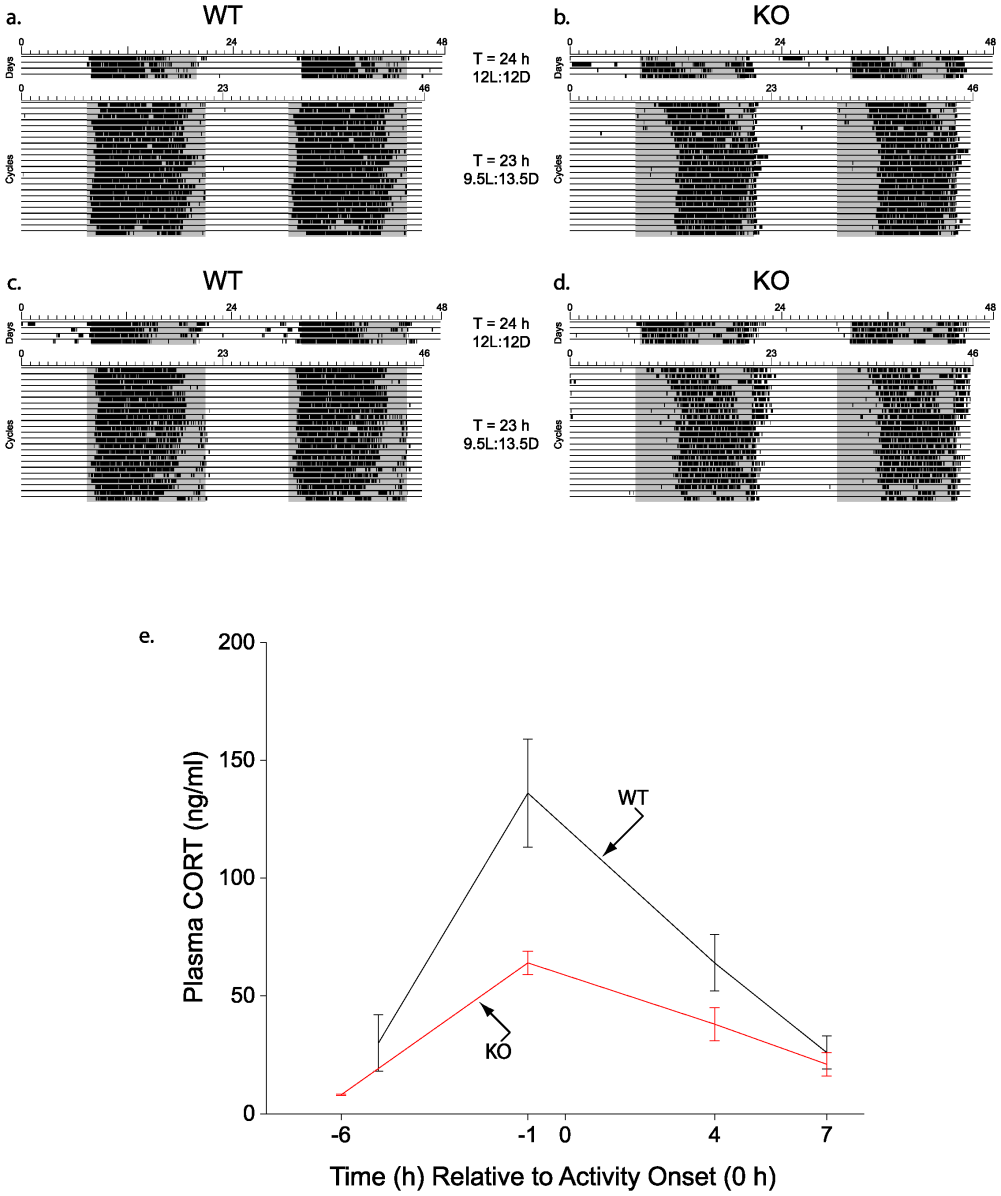
Activity and corticosterone levels of wild type and 5-HT_1B_ knockout mice in 9.5L:13.5D conditions. Wheel-running activity records double-plotted in the standard manner, with each day's activity plotted beneath and after the previous day's activity (shaded region represents dark). Examples of activity of wild type (WT; a & c) and 5-HT_1B_ knockout (KO; b & d) mice transferred from 12L:12D (T = 24 h) to 9.5L:13.5D (T = 23 h) conditions. Animals re-entrained to the short-day 23 h cycle although wheel-running activity onsets, relative to light offset, were significantly more delayed in KO animals compared to WT animals in the 9.5L:13.5 D conditions (p<0.001). (e) Plasma corticosterone (CORT) levels exhibited a diurnal rhythm with peak values observed one hour before activity onset (−1 h time point); the peak plasma CORT value for KO animals was significantly less than peak plasma CORT value of WT mice (p<0.01). 9–13 plasma samples/time point.

To test the hypothesis that altered entrainment of the circadian rhythm of wheel-running activity to the day/night cycle also alters the diurnal rhythm of plasma CORT, wild type (WT) (n = 23) and 5-HT_1B_ KO (n = 23) mice were housed under 12L:12D conditions for several weeks and wheel-running activity was recorded to confirm that all animals were entrained before lighting conditions were changed. In mice housed under 12L:12D conditions, photic information preceding light offset is used to phase delay and reset the SCN clock each day [17]. All mice of both genotypes entrained to the 12L:12D cycle with activity onsets at lights off as expected. After lighting conditions were changed to 9.5L:13.5D (a short-day 23 h cycle), all animals re-entrained their wheel-running activity to the new lighting regimen (Figs. 1a–d). In mice housed under 9.5L:13.5 D conditions, light onset is used to reset the SCN clock by evoking phase advances each day because the endogenous period of the mouse SCN (approximately 23.6 h) is now greater than the period of the day/night cycle. Therefore activity onsets of mice are typically delayed relative to lights off, allowing a greater portion of the animal's late subjective night to be coincident with the dawn light [17]. After ≈23 cycles under 9.5L:13.5D conditions, the phase angle of entrainment (Ψ, activity onset relative to light offset) of WT and 5-HT_1B_ KO mice was delayed compared to Ψ under 12L:12D conditions as expected. However, Ψ of 5-HT_1B_ KO mice was delayed 5.3±0.2 h (mean ± SEM) (range 4.2–7.9 h), significantly more than Ψ of WT animals, which were delayed only 2.3±0.4 h (range 0.4–6.0 h; p<0.001) (Figs. 1a–d).

It is well documented that the diurnal rhythm of CORT peaks shortly prior to activity onset [20]. Therefore plasma CORT levels were assayed at four time points relative to activity onset (defined as time 0) without regard to when in the day/night cycle activity onsets occurred. CORT levels were determined at 6 h (or 5 h) and 1 h before activity onset (−6 h, −5 h, and −1 h time points), and at 4 h and 7 h after activity onset (4 h, 7 h time points). The peak plasma CORT value for both the WT and 5-HT_1B_ KO mice was observed at the −1 h time point although the peak CORT value for KO mice was significantly less than that of the WT mice under the 9.5L:13.5D conditions (KO, 64±5 ng/ml, n = 10, vs WT, 136±23 ng/ml, n = 9; p <0.01) (Fig. 1e). The Ψ of the subset of WT animals sampled at the −1 h time point was 0.6±0.04 h (range 0.4–0.75 h, n = 9), significantly different than the Ψ of the subset of KO animals sampled at the −1 h time point (4.7±0.06 h, range 4.5–5.1 h, n = 10) (p<0.0001). These data suggest that 5-HT_1B_ KO mice generate a diurnal rhythm of plasma CORT synchronized with the concurrently assessed activity rhythm, similar to WT mice, despite the altered phase relationship of the activity rhythm to the L∶D cycle. However, the amplitude of the diurnal rhythm of plasma CORT of 5-HT_1B_ KO mice with a highly delayed phase angle of entrainment is significantly attenuated and is reduced ≈50% compared to controls. An alternative interpretation of the data is that 5-HT_1B_ KO mice are simply unable to generate a normal amplitude in the diurnal CORT rhythm under any conditions.

### Experiment 2: CORT rhythms in 5-HT_1B_ KO and WT mice under 12L:12D conditions

To examine the possibility that the attenuated peak in the diurnal rhythm of plasma CORT observed in 5-HT_1B_ KO mice under 9.5L:13.5D 23 h lighting conditions was due to an inability of these mice to generate a plasma CORT rhythm with normal amplitude, we next examined wheel-running activity in WT (n = 18) and 5-HT_1B_ KO (n = 15) mice maintained under 12L:12D conditions for several weeks. WT and 5-HT_1B_ KO mice entrained their wheel-running behavior to the 12L:12D conditions with wheel-running activity onsets initiated at light offset as expected (data not shown).

**Figure 2:**
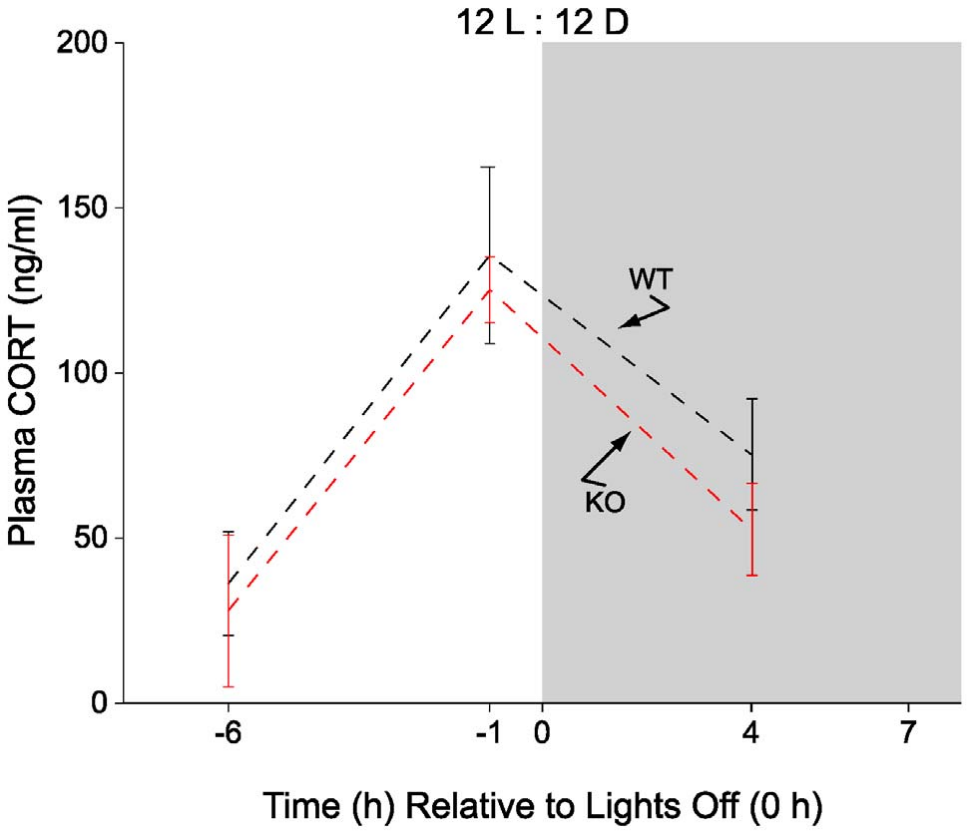
Plasma CORT levels of wild type and 5-HT_1B_ knockout mice in 12L:12D conditions. Circulating CORT values of wild type (WT) and 5-HT_1B_ knockout (KO) animals, sampled at three time points under 12L:12D conditions, exhibited a diurnal rise with peak CORT values at −1 h. CORT values of WT and KO mice were similar at each of the time points sampled. The −6 h and +4 h time points were significantly less than −1 h time point (p<0.05) (4–7 animals/time point; shaded region represents dark).

**Figure 3:**
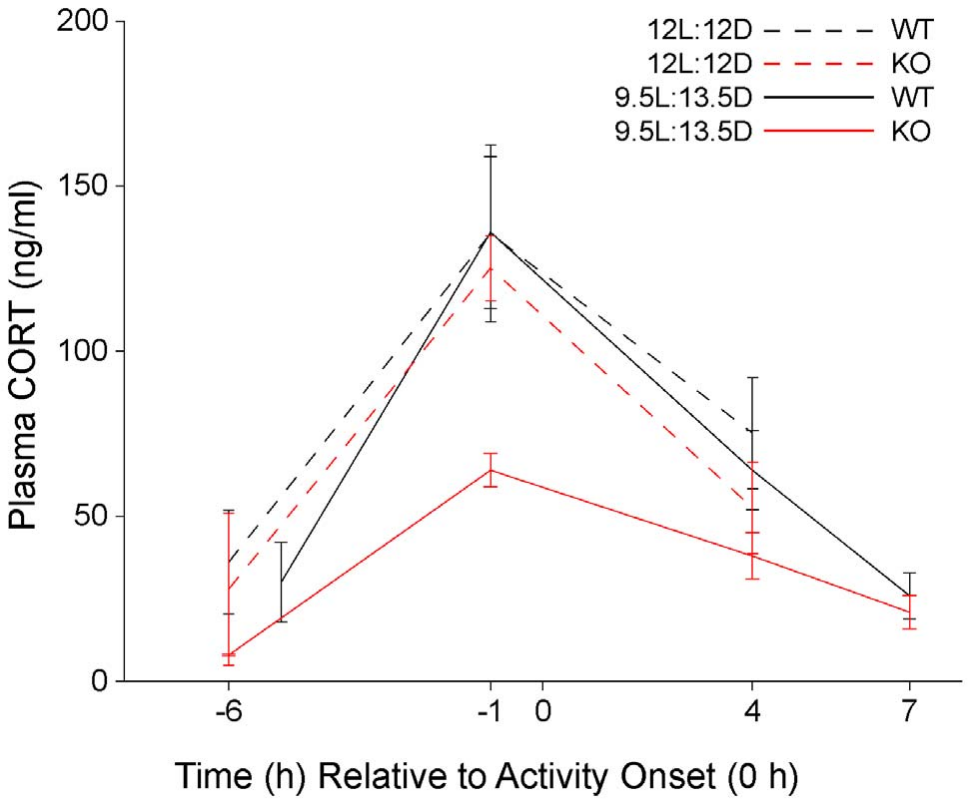
Compilation of plasma CORT data from Experiments 1 & 2 normalized relative to activity onsets. Peak CORT values at 1 h before activity onset of wild type (WT) mice maintained under 12L:12D and 9.5L:13.5D and 5-HT_1B_ knockout (KO) mice maintained under 12L:12D conditions were similar and significantly greater than −1 h CORT values of KO mice maintained under 9.5L:13.5 D conditions (p<0.01).

After ≈21 days in 12L:12D conditions, CORT levels were determined at 6 h and 1 h before activity onset and 4 h after activity onset with 4–7 mice of each genotype sampled at each time point. As illustrated in Figure 2, basal and peak plasma CORT values of WT and 5-HT_1B_ KO mice were very similar with no significant difference observed between genotypes at any of the time points examined. Moreover, peak plasma CORT values of WT mice under 12L:12D conditions were indistinguishable from peak plasma CORT values of WT mice observed under 9.5L:13.5D conditions (12L:12D, 136±27 ng/ml, n = 6 vs 9.5L:13.5D, 136±23 ng/ml, n = 9) (Fig. 3).

These results indicate that WT mice entrained to either 12L:12D or 9.5L:13.5D conditions with activity onsets at lights off (this experiment) or slightly delayed relative to light offset (0.6 h, Experiment 1), generate a diurnal rhythm of plasma CORT with similar amplitude. Moreover, 5-HT_1B_ KO mice entrained to the 12L:12D cycle with activity onsets at light offset, similar to WT mice, generate a diurnal rhythm of plasma CORT of normal amplitude. Thus the diurnal CORT rhythm is not affected by the absence of the 5-HT_1B_ receptor. The peak of the plasma CORT rhythm of 5-HT_1B_ KO in 12L:12D is significantly greater than that generated by 5-HT_1B_ KO mice with activity onsets that are highly delayed as observed in Experiment 1 under 9.5L13.5D conditions.

### Experiment 3: CORT rhythms in 5-HT_1B_ KO and WT mice under constant dark conditions

Plasma used for the −1 h CORT determination in Experiment 1 was collected from WT mice with activity onsets that were initiated approximately 30 min after light offset whereas the 5-HT_1B_ KO mice that were sampled at the −1 h time point had activity onsets delayed over 4 h. Thus, blood collected at the −1 h time point from KO mice was from animals that were in the dark at the time of collection whereas the WT mice that were sampled at −1 h were in the light when blood was collected. Light exposure increases plasma CORT in humans and rodents [26], [27], [49]–[52] and in mice this response to light is independent of ACTH [26], [27]; light therefore can contribute to activation of the adrenal gland and CORT secretion. These findings raise the possibility that the peak plasma CORT values in Experiment 1 may have been influenced by the lighting condition at the time of blood collection. To test the effect of light on the rhythm of plasma CORT, we examined peak plasma CORT levels collected from mice that were in the dark at the time of sampling to compare with peak plasma CORT values from mice that were in the light at the time of sampling. WT mice (n = 49) were maintained in 12L:12D for 23 days with wheel-running activity continuously recorded. On day 24 a group of mice (n = 24) was placed into constant dark (DD) conditions. Two and three days later, blood was collected at three time points (−4 h, −1 h, and +3 h relative to activity onset) for plasma CORT determination in both groups.

**Figure 4:**
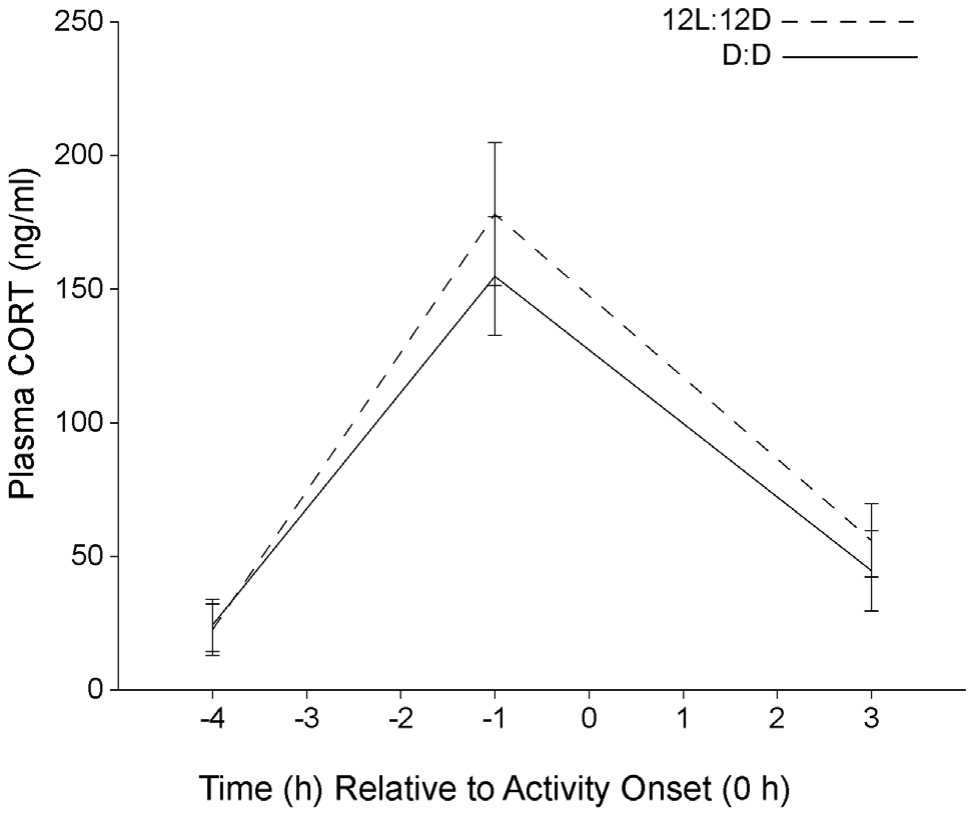
Plasma CORT values in wild type mice under 12L:12D and constant dark conditions. Circulating CORT values exhibited a circadian rhythm in wild type mice under constant dark (DD) conditions, with values similar to the diurnal rhythm values of plasma CORT observed under 12L:12D conditions.

Plasma CORT values of WT animals in 12L:12D and DD were similar at all three time points sampled (Fig. 4). The peak plasma CORT value at −1 h from animals in DD appeared to be slightly reduced (155±23 ng/ml, n = 12, DD vs 178±28 ng/ml, n =  9, LD) although the difference was not significant. These data confirm the circadian nature of the rhythm in CORT secretion as it persists under DD conditions. Moreover, these data do not support the hypothesis that peak plasma CORT values were reduced in 5-HT_1B_ KO mice in Experiment 1 because plasma was collected from animals in the dark.

### Experiment 4: Phase angle of entrainment vs peak plasma CORT

To further explore the relationship between the phase angle of entrainment of wheel-running activity rhythm to the L∶D cycle and the diurnal rhythm of circulating CORT, activity was recorded from WT (n = 52) and 5-HT_1B_ KO (n = 68) mice in four independent experiments similar to Experiment 1. In each experiment mice were maintained under 12L:12D conditions for several weeks and then the lighting conditions were changed to 9.5L:13.5D. After 21–23 cycles under 9.5L:13.5D conditions, similar to the number of cycles after which peak plasma CORT levels were determined in Experiment 1, blood was collected from animals 1 h before activity onset and CORT values were determined. Collectively, activity onsets ranged from 0.0 h to 6.7 h after light offset and −1 h plasma CORT values were determined from a total of 64 animals (see Methods for plasma sampling restrictions). The −1 h plasma CORT values in Experiment 1 are included in this analysis providing a total of 83 plasma CORT values.

**Figure 5:**
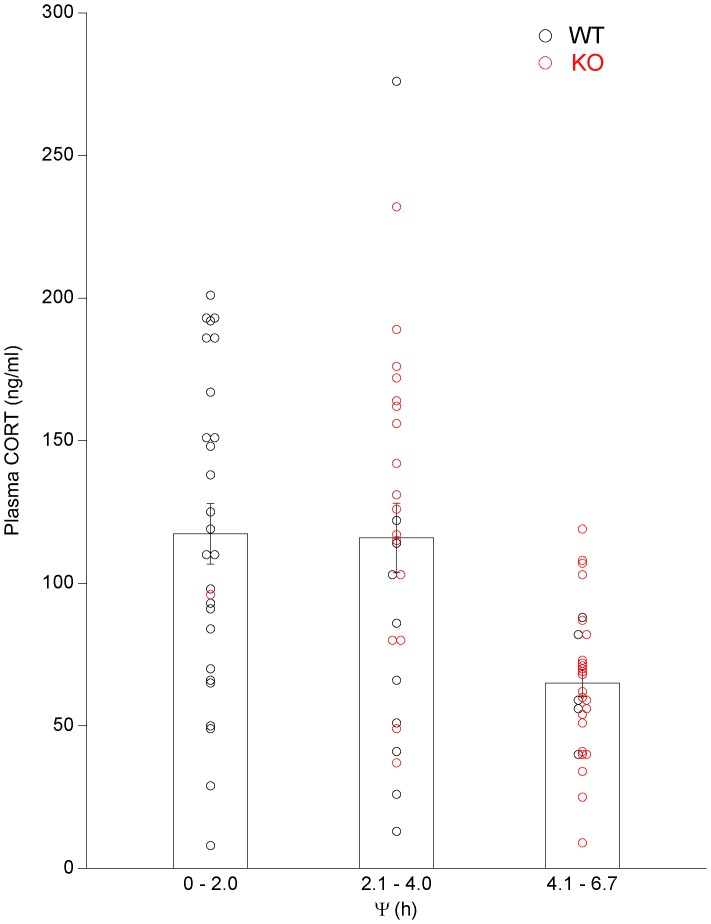
Peak plasma CORT levels of wild type and 5-HT_1B_ knockout mice in 9.5L:13.5D. Plasma CORT values, determined 1 h prior to activity onset after 21–23 cycles in 9.5L:13.5D for wild type (WT) and 5-HT_1B_ knockout (KO) animals, assembled into groups based on the animals phase angle of entrainment (Ψ, activity onset relative to light offset). Peak CORT levels are significantly attenuated in animals with Ψs delayed>4 h after light offset (p<0.01); (n) = 27–29 animals/group.

**Figure 6:**
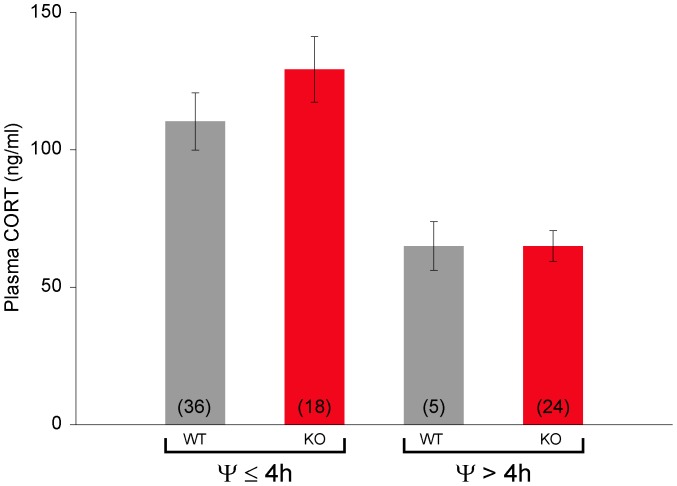
Peak plasma CORT levels are reduced in animals with delayed activity onsets in 9.5L:13.5D. Peak plasma CORT values for WT and KO animals entrained with activity onsets ≤4 h or>4 h after light offset. Peak plasma CORT values are dependent on Ψ (p<0.0001) but not genotype (p>0.05).

The relationship between −1 h plasma CORT values and Ψ was analyzed by grouping animals into approximately two-hour bins based on the time of their activity onsets relative to light offset (0–2.0 h, n = 27; 2.1–4.0 h, n = 27; and 4.1–6.7 h, n = 29). A one-way ANOVA indicated a significant difference between the three groups (p<0.0001) and a post-hoc analysis indicated plasma CORT values of animals phase delayed 4.0–6.7 h were significantly lower than plasma CORT values in animals phase delayed 0–2.0 h (p<0.01) or 2.1–4.0 h (p<0.01) whereas plasma CORT values for the groups 0–2.0 h and 2.1–4.0 h were not significantly different (Fig. 5). When mouse genotype was compared to plasma CORT values for animals with Ψ≤4.0 h and Ψ>4.0 h, there was a significant effect of Ψ (two-way ANOVA p<0.0001) but not genotype (Fig. 6).

To control for the possibility that the peak in the diurnal rhythm of plasma CORT was slightly shifted and therefore missed in Experiment 1 when blood was assayed at the −6 h, −1 h, 4 h, and 7 h time points, blood samples were collected at −3 h and +1 h from a subset of animals with activity onsets delayed 5.3±0.3 h (n = 9; range 4.3–6.7 h). Plasma CORT levels were low at the −3 h time point (25±9 ng/ml, n = 9) and the 1 h time point (31±9 ng/ml, n = 9) indicating that by sampling at the −1 h time point we did not miss a shifted peak in plasma CORT in animals with a highly delayed Ψ. Taken together, the data indicate that the peak of the diurnal rhythm in plasma CORT is significantly reduced in animals with a phase angle of entrainment delayed>4 h after light offset.

### Experiment 5: Diurnal CORT rhythms in WT mice under 8L:14D conditions

**Figure 7:**
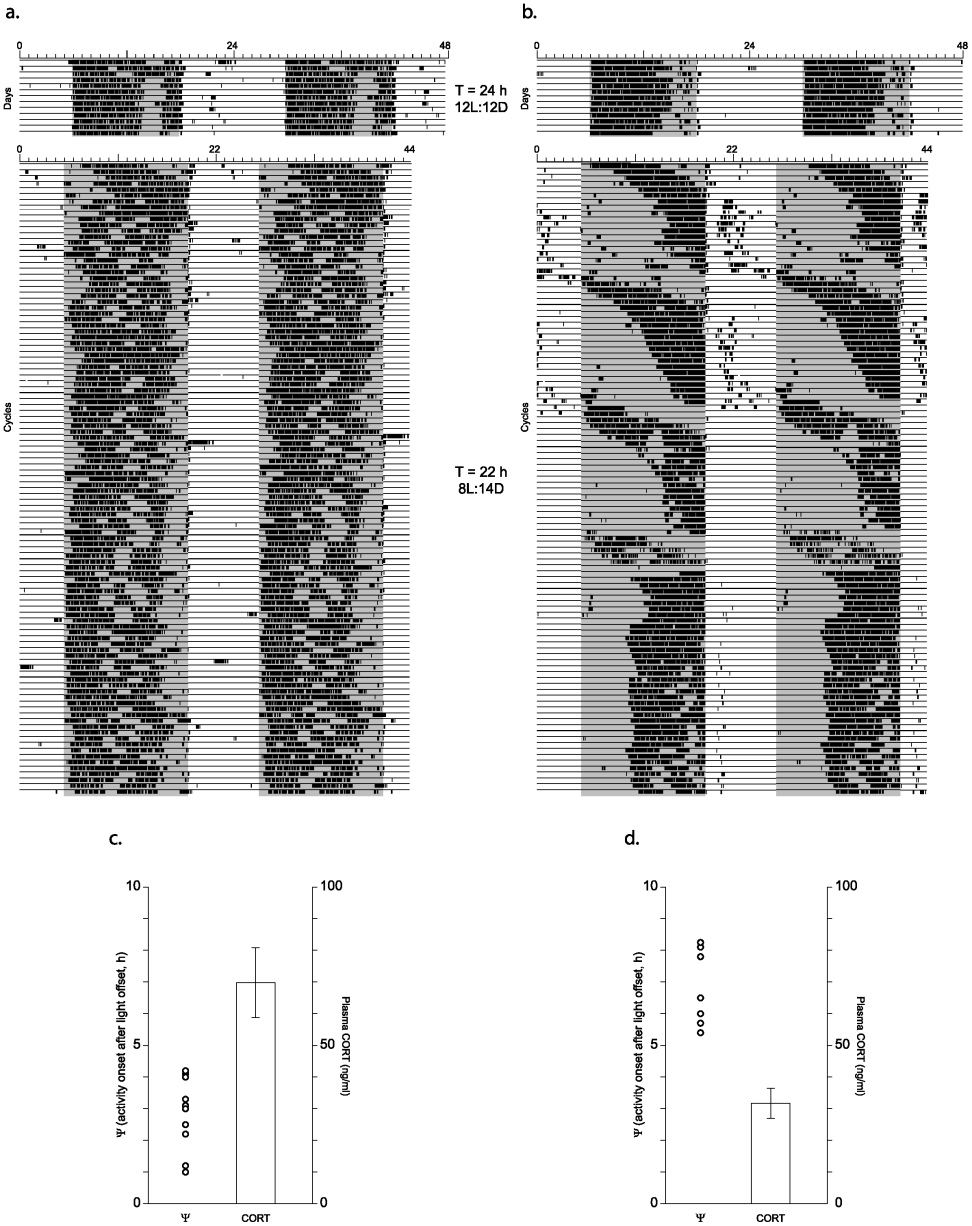
Wheel-running activity records and peak plasma CORT values of wild type mice in 8L:14D. (a) An animal that re-entrained to 8L:14D quickly with activity onsets <1 hour delayed after light offset. (b) An example of an animal that required ≈2 months to re-entrain to 8L:14D with activity onsets delayed>5 h after light offset. (c) Peak plasma CORT values of mice with Ψs≤4.2 h delayed (n = 12) as illustrated by example in panel (a). (d) Peak plasma CORT values of mice with Ψs≥5.4 h delayed (n = 7) as illustrated by the example in panel (b). CORT values of animals with a Ψ≥5.4 h delayed were significantly less than CORT values of animals with a Ψ≤4.2 h delayed (p<0.03).

The findings provided above indicate that the reduction in the peak of the diurnal rhythm of circulating CORT is related to the phase angle of entrainment to the L∶D cycle rather than the lack of the 5-HT_1B_ receptor. To further test whether peak plasma CORT values are reduced in WT mice with a delayed Ψ, we maintained WT mice under an 8L:14D (short-day) T = 22 h cycle and recorded wheel-running activity. As in the experiments described above, animals (n = 36) were first housed under 12L:12D conditions for several weeks and then the lighting conditions were changed to 8L:14D (T = 22 h). The experiment was terminated after animals had been in 8L:14D for approximately 107 cycles (Figs. 7a & 7b).

Most animals re-entrained after transfer to the 8L:14D conditions within a few cycles (Fig. 7a) although some animals required weeks or months to re-entrain (Fig. 7b) and two animals had not re-entrained to the 8L:14D cycle when the experiment was terminated (data not shown). The phase angle of entrainment of the WT mice observed under the 8L:14D conditions was quite variable, with activity onsets ranging from approximately light offset to over 8 h after light offset (Figs. 7c & 7d). Moreover, activity onsets in many cases had a high degree of day-to-day variability rendering it difficult to determine an accurate time for blood collection in many of the animals; blood samples were not collected from these animals. Thus, −1 h plasma CORT levels were determined in 19 of the 36 animals, 12 of which had a Ψ≤4.2 h (range 1.0–4.2 h; Fig. 7c) and 7 that had a Ψ≥5.4 h (range 5.4–8.25 h; Fig. 7d). The −1 h plasma CORT values were significantly lower in the WT animals with a Ψ≥5.4 h (31.7±4.8 ng/ml, n = 7) compared to the WT animals with a Ψ≤4.2 h (69.8±11.0 ng/ml, n = 12; p<0.03). It is noteworthy that the −1 h plasma CORT levels were unusually low in both populations although a cause other than entrainment to the 22 h day could not be determined. Nevertheless, even in the context of this reduced CORT level, animals with greatly delayed activity onsets expressed lower plasma CORT values than the animals with activity onsets that were phase delayed ≤4.2 h, consistent with the findings from highly phase delayed animals in the preceding experiments. These data taken together indicate that a highly delayed phase angle of entrainment of the circadian activity rhythm to the day/night cycle (i.e., more than ≈4 delayed) is associated with a pronounced reduction in the diurnal rhythm of circulating CORT.

### Experiment 6: ACTH challenge, and clock gene expression in WT mice in 8L:14D

In this experiment we began to explore the potential mechanisms underlying the profound reduction in the peak amplitude of the daily rhythm of plasma CORT in animals with activity onsets delayed more than 4 h after light offset. The normally high amplitude diurnal rhythm of plasma CORT is largely due to a coincident rhythm in adrenal sensitivity to ACTH [21]. If the rhythm in adrenal sensitivity to ACTH becomes out of phase with the SCN-driven rhythm of ACTH release, a reduction in adrenocortical CORT synthesis might be the consequence. Such a misalignment between adrenal and SCN oscillations could provide a possible mechanism for the reduced peak plasma CORT levels described above in animals with considerably delayed phase angles of entrainment (i.e., onsets>4 h delayed). In this experiment we tested a specific corollary of this general hypothesis, that the adrenal rhythm of sensitivity to ACTH remained aligned with light offset and not with the SCN-driven rhythms.

Another group of 36 WT mice were subjected to the same 22 h lighting regimen as those in Experiment 5. Animals were maintained in running-wheel cages and after several weeks in 12L:12D, the lighting conditions were changes to 8L:14D for ≈110 cycles. At the termination of the experiment adrenal glands and brains were collected for subsequent analysis.

Similar to the animals maintained in 8L:14D in Experiment 5, most animals re-entrained to the 8L:14D conditions within a few cycles and some required many weeks or months to re-entrain; 3 mice had not re-entrained to the 8L:14D conditions when the experiment was terminated after ≈105 days (data not shown). The phase angle of entrainment was again quite variable among these animals, ranging from 0 h (at light offset) to ≈8 h after light offset and the day-to-day variability in activity onsets was considerable in many animals.

**Figure 8:**
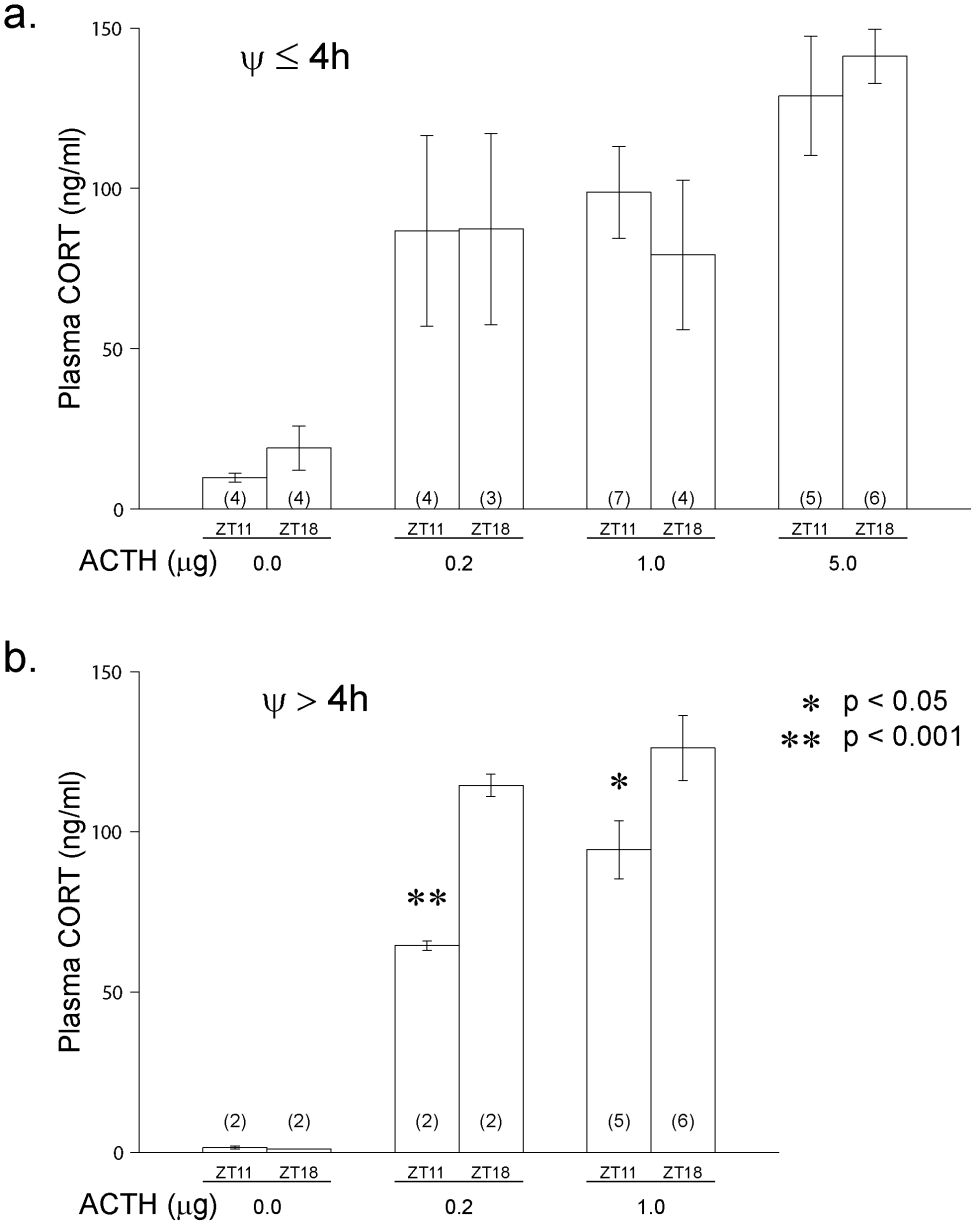
Circulating CORT levels after ACTH challenge. Mice in 8L:14D entrained with (a) Ψs≤4 h delayed and (b) Ψs>4 h delayed were pretreated with dexamethasone (100 µg/kg ip) and 2 h later were challenged with either vehicle or ACTH (0.2, 1.0 or 5.0 µg ACTH/mouse ip). Blood samples were collected 20 min after ACTH injection at either ZT 11 or ZT 18. All CORT values after ACTH injection were significantly greater than CORT values following vehicle injection (p<0.01). ZT11 significantly different from ZT18, * = p<0.05; ** = p<0.001. (n) = number mice/group.

To test adrenal responsiveness to ACTH stimulation, mice were challenged with exogenous ACTH after a dexamethasone block of endogenous ACTH. Between cycles 71–78 in 8L:14D, animals were treated with dexamethasone (100 µg/kg) to suppress ACTH secretion and they were then challenged with two or three doses of ACTH at two time points; blood was collected at 1 h before light offset or Zeitgeber Time 11 (ZT11; ZT12 is defined as light offset) and 6 h after light offset (ZT18). As expected, ACTH injection caused a dose-dependent increase in circulating CORT levels at ZT11 and ZT18 (Figs. 8a & 8b). However, the adrenal response to 0.2 µg and 1.0 µg of ACTH injected at ZT18 was significantly greater than at ZT11 in animals with a Ψ>4 h delayed (range 5.0–8.4 h) (Fig. 8b); the adrenal responses to ACTH injections at ZT11 and ZT18 in animals with Ψ<4 h delayed (range 0.3–3.9 h) were not significantly different at any dose of ACTH administered (Fig. 8a). These data indicate that the rhythms of adrenal responsiveness to ACTH in the two populations of mice were in different phases relative to light offset and consequently were likely to be in phase with the SCN-driven locomotor activity onset.

At the termination of the experiment, adrenal glands and brains were collected from animals that had maintained relatively stable entrainment throughout; tissues were collected at ZT12 and ZT18 from 8 animals that had Ψ's<4 h after light offset (range 0.0–1.7 h) and from 6 animals that had Ψ's>4 h after light offset (range 5.7–8.0 h).

**Figure 9:**
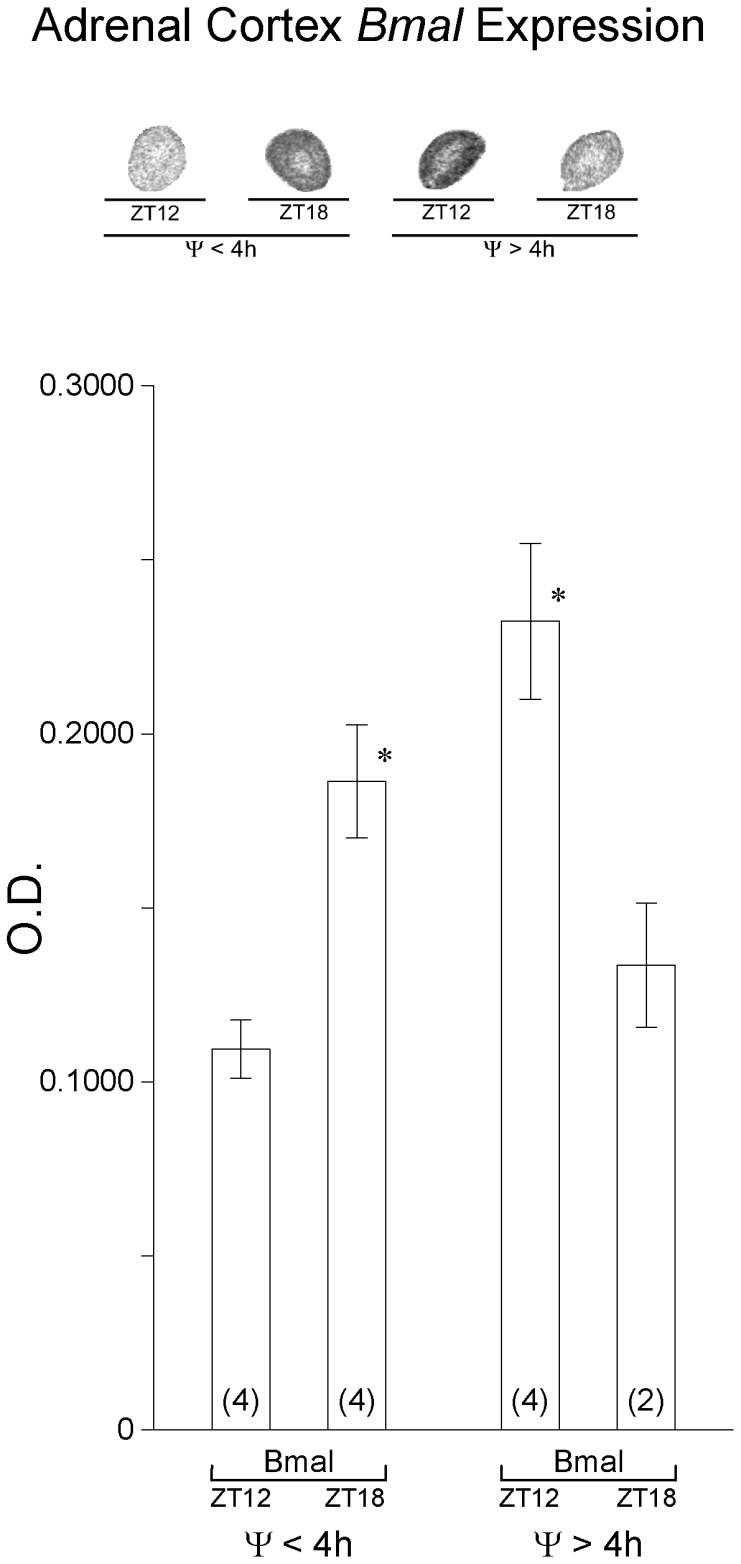
Clock gene expression in the adrenal cortex. (top) Representative autoradiogram images of *bmal1* expression in the adrenal cortex measured by *in situ* hybridization for animals in 8L:14D entrained with Ψs≤4 h delayed and Ψs>4 h delayed and killed at ZT 12 and ZT 18. Quantification shows that *bmal1* expression is significantly greater at ZT 18 in animals with Ψs≤4 h delayed whereas *bmal1* expression in significantly greater at ZT 12 in animals with Ψs>4 h delayed indicating the adrenocortical molecular rhythms are in different phases relative to light offset.

Clock genes are rhythmically expressed in the adrenal cortex; under 12L:12D conditions, *bmal1* expression is at its nadir at ZT12 [53]. We examined *bmal1* expression to assist in estimating the phase of the adrenal gland in animals with activity onsets significantly delayed after light offset. If the adrenal were aligned with light offset, the expression of *bmal1* would be predicted to be similar in both groups of mice, with *bmal1* expression greater at ZT18 than at ZT12. Contrary to this prediction, *bmal1* expression appeared out of phase between the two groups of mice (Fig. 9). At ZT18 adrenal cortex *bmal1* expression was significantly greater than that detected at ZT12 in mice with Ψ's<4 h delayed whereas the opposite was observed in mice with Ψ's>4 h delayed (Fig. 9). These data are consistent with the interpretation that the adrenocortical rhythm in clock gene expression is in phase with activity onset rather than with light offset.

**Figure 10:**
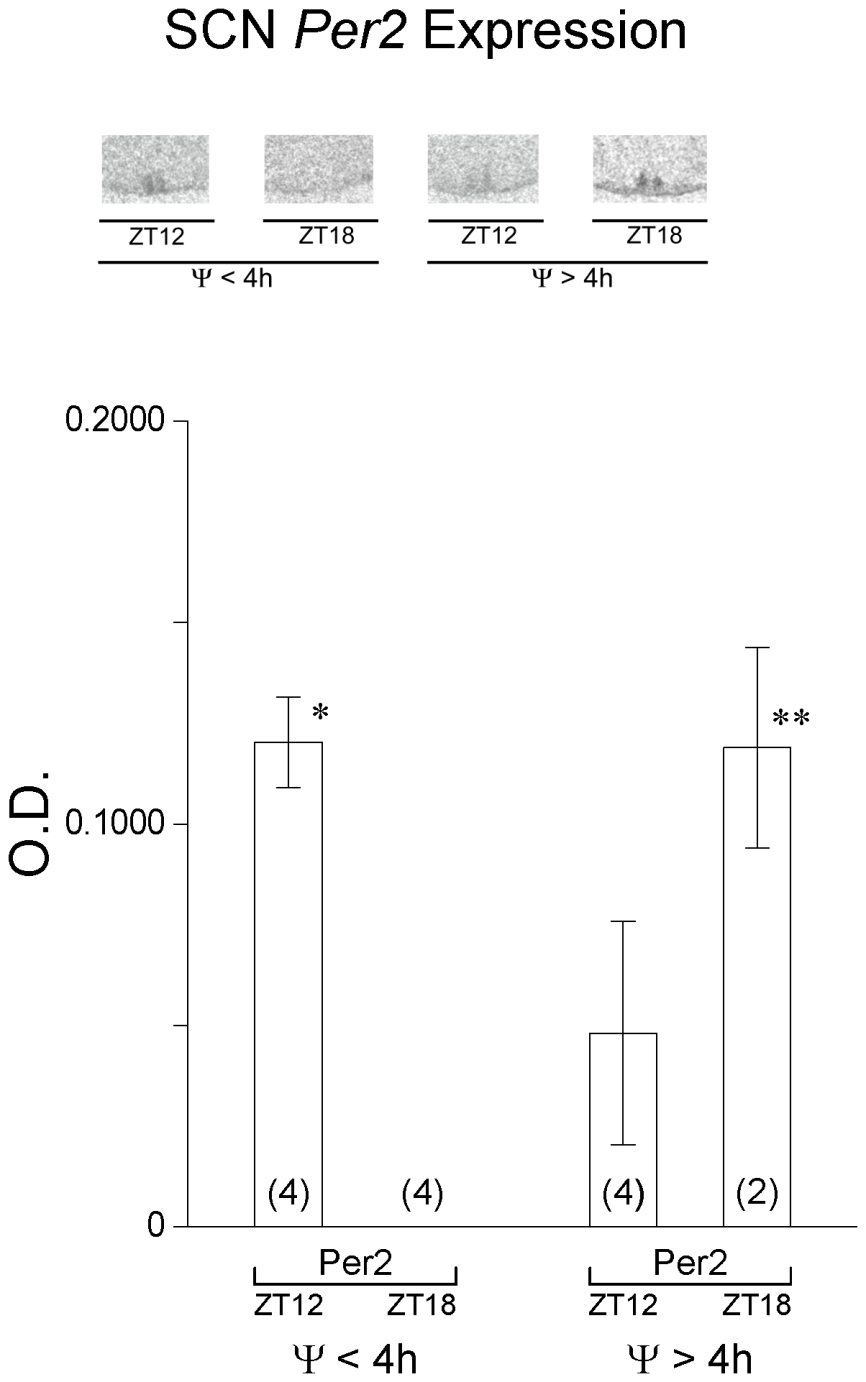
Clock gene expression in the suprachiasmatic nucleus. (top) Representative autoradiogram images of *per2* expression in the suprachiasmatic nucleus (SCN) measured by *in situ* hybridization for animals in 8L:14D entrained with Ψs≤4 h delayed and Ψs>4 h delayed and killed at ZT 12 and ZT 18. Quantification shows that *Per2* expression is significantly greater at ZT 12 vs ZT18 in animals with Ψs≤4 h delayed whereas in animals with Ψs>4 h delayed, *per2* expression is significantly greater at ZT18 vs ZT12 indicating that the SCN clock gene rhythms are in different phases in these two populations of animals relative to light offset.

Clock gene expression was also examined in the SCN of these animals. SCN *per2* expression is known to peak at ZT12 and decrease sharply by ZT18 under 12L:12D conditions [53]. Thus, *per2* expression is particularly valuable in estimating phase of the SCN in the animals killed at ZT12 and ZT18. In animals with activity onsets close to light offset, SCN *per2* expression was significantly higher at ZT12 compared to ZT18 (Fig. 10). The opposite was noted for animals with Ψ's>4 h delayed after light offset; SCN *per2* expression was significantly increased at ZT18 compared to expression levels at ZT12 (Fig. 10). These data strongly suggest that the onset of wheel-running activity is in phase with the SCN clock gene oscillation despite the altered phase relationship to the L∶D cycle. Taken together, the data from this experiment do not provide support for the hypothesis that the reduction in the amplitude of the daily rhythm of plasma CORT is a result of the adrenal gland circadian clock gene oscillation being aligned with light offset in mice with Ψ's>4 h. The data do suggest that adrenal oscillations are in phase with locomotor activity, which is driven by the SCN.

## Discussion

The principal novel finding reported in this study is that altered entrainment of the SCN biological clock to the day/night cycle can severely attenuate the daily rise in circulating corticosterone. The effect of altered entrainment to the day/night cycle on CORT rhythm amplitude was evident when the onset of the circadian activity rhythm of 5-HT_1B_ KO and WT mice was delayed more than ≈4 hours relative to light offset. However, because elimination of the 5-HT_1B_ receptor renders the SCN circadian system of the KO animals less sensitive to light input via the RHT [12], [17]–[19] the phase relationship is altered. Under light cycles that are both “short-day” (winter-like) and T<24 h, the 5-HT_1B_ KO mouse activity rhythm is more likely to be shifted to provide greater daily light exposure sufficient to reset the SCN clock [17]. Thus, attenuation of the daily plasma CORT rhythm is more prevalent in animals with altered 5-HT neurotransmission compared to WT mice maintained under identical 23 h short-day conditions.

Basal CORT levels and the acute response of the HPA axis of male 5-HT_1B_ KO mice to mild stress have been examined previously and were found comparable to those of WT mice [54]. We extend those findings in the present study by showing that 5-HT_1B_ KO mice generate a daily rhythm of plasma CORT when housed under 12L:12D conditions and a circadian rhythm of plasma CORT when maintained under DD conditions, and that both are comparable to the plasma CORT rhythms of WT animals. These data reinforce the interpretation that the highly delayed phase angle of entrainment to the day/night cycle, rather than the lack of the 5-HT_1B_ receptor, is associated with the altered plasma CORT rhythm observed under 9.5L:13.5D, T = 23 h conditions in this study. Moreover, WT mice with a Ψ more than 4 h delayed while maintained under 8L:14D, T = 22 h conditions also exhibit an attenuated daily rhythm of plasma CORT. It appears that the phase angle of entrainment is the critical factor with regard to the amplitude of the daily rhythm in circulating CORT.

### Entrainment mechanisms

Under the experimental conditions employed in this study, mice use daily advance phase shifts to entrain to the 22 h or 23 h day/night cycles since the period of their endogenous SCN clock (τ = 23.6 h; established previously under DD conditions [17]) is greater than the period of these environmental L∶D cycles. The magnitude and sign of the daily light-induced phase shift required for photic entrainment is determined by the difference between the period of the endogenous SCN oscillator (τ) and the period of the L∶D cycle (T) (daily phase shift = τ – T). Light-induced advance phase shifts are generated in the late subjective night and therefore when T = 23 h, light onset each day is used to reset the SCN clock of mice. 5-HT_1B_ KO mice require a greater duration of light exposure to compensate for their reduced responsiveness to light. Thus, the phase of their circadian cycle that is sensitive to light is shifted (delayed) so that a greater portion of their late subjective night is coincident with the morning light [17].

Measurements of the period of the human circadian pacemaker have yielded inconsistent results, with period length estimates as long as 25 h [55]. Recent work suggests the period of the human circadian clock may be closer to 24 h than to 25 h [56] but nevertheless there is widespread agreement that the period of the human circadian clock is >24 h [57]. Entrainment mechanisms in humans are considered similar to those of rodents with the exception that thresholds appear higher in people. In addition, it has been reported that humans may not have a dead zone during the subjective day although the long duration (≈7 h) of the light pulse administered in that study may have obscured the dead zone's presence [58]. Thus humans must use daily advance phase shifts of the circadian clock as the primary photic cue for entrainment to the 24 h environmental day/night cycle, generated by exposure to light at dawn. A reduction in the sensitivity of the human circadian system to light (or an abnormal lengthening of the endogenous circadian period) would be predicted to produce a delayed phase angle of entrainment such that a greater portion of the late subjective night is exposed to the morning light, similar to the 5-HT_1B_ KO mice described in the present study and reported previously [17].

### Is a reduced diurnal rhythm of plasma CORT noteworthy?

It has become increasingly evident that the daily rhythm in corticosterone may represent an important temporal signal regulating rhythmic gene expression in the brain and major organ systems of the body. Corticosterone is a potent transcriptional regulator that is critical for the rhythmic expression of the clock gene *per2* in the oval nucleus of the bed nucleus of the stria terminalis and central nucleus of the amygdala [28], [59]–[61]. The daily CORT rhythm regulates μ-opioid receptor expression in the brainstem [29] and drives *tryptophan hydroxylase-2* expression in serotonergic neurons in the dorsal and median raphe [30], [31]. It has been suggested that the CORT-induced increase in 5-HT synthesis in the median raphe nucleus may provide a feedback loop to the SCN, which is devoid of glucocorticoid receptors, and thereby contribute to regulating the sensitivity of the SCN to light [62], [63].

Rhythmic glucocorticoid signaling has also been implicated in the synchronization of subsidiary circadian oscillators in peripheral tissues [32]. Major organ systems including lungs, heart, kidney, liver, and pituitary all contain autonomously rhythmic circadian clocks that are dependent for their synchronization at the tissue level on temporal cues from the SCN [20]. Coordination of rhythmic liver metabolism is particularly dependent on the daily plasma CORT rhythm [64]. Thus, a dampening of the diurnal plasma CORT rhythm may have wide-ranging effects both on neuronal function of key limbic forebrain structures and transmitter systems that affect mood and the temporal organization of peripheral tissues critically important for whole body metabolism [36]–[39].

Circadian regulation of the HPA axis is mediated in large part by glucocorticoid activation of both the high affinity mineralocorticoid receptor and the lower affinity glucocorticoid receptor (GR) in the brain. In peripheral organs CORT exerts its effects largely by activation of the GR. Due to their low affinity for CORT, GRs are significantly activated only by relatively high levels of circulating CORT, such as during the daily rise or after stress [36], [65]. However, even at plasma levels attained at the peak of the diurnal CORT rhythm, GR occupation may only be about 50% (74). The nuclear translocation of GR after activation differs in a dose-dependent manner in different regions of the brain [66], [67]. Thus a 50% reduction in the amplitude of the peak of the daily plasma CORT rhythm would result in relatively low GR occupancy in general and the reduced plasma CORT peak could also differentially affect diverse regions in the brain.

### How does altered entrainment dampen the daily rhythm of plasma CORT?

It is often stated that the robust circadian rhythm in plasma CORT is derived from rhythms in the HPA-axis: i.e., that the SCN circadian clock drives rhythmic CRH secretion that in turn drives rhythmic ACTH release from the anterior pituitary resulting in rhythmic CORT synthesis and secretion from the adrenal cortex [68]. Although circadian rhythms have been documented at all levels of the HPA-axis, there is increasing evidence indicating that rhythms of CRH and ACTH are not responsible for the observed daily rhythm in plasma CORT [69]. For example, when *Crh^−/−^* mice are given a constant infusion of CRH (producing no diurnal change in plasma ACTH), a diurnal rhythm in plasma CORT is rapidly restored indicating that diurnal variation of CRH and ACTH secretion are not necessary to drive rhythmic secretion of CORT from the adrenal [70]. Similarly, constant ACTH replacement in hypophysectomized rats also generates a diurnal variation in plasma CORT demonstrating again that the normally low amplitude rhythm in plasma ACTH is not necessary to drive rhythmic CORT secretion [22]. Thus, although there are daily rhythms in CRH and ACTH these are not responsible for the daily plasma CORT rhythm.

The primary mechanism underlying the rhythmic synthesis and secretion of CORT appears to be intrinsic to the adrenal. The adrenal has long been known to be capable of self-sustained oscillations in steroid biogenesis when supplied with constant levels of ACTH [71], [72]. The rhythm in CORT production results from diurnal variation in adrenal responsiveness to ACTH [21], [73], [74]. These early studies have been strengthened by more recent investigations showing that the adrenal possesses a circadian clock mechanism, like most tissues and organs in the body [75]. The circadian clock in the adrenal gates the responsiveness to circulating ACTH [76]–[80]. This is accomplished at least in part, by the adrenal circadian clock regulation of genes involved in the control of CORT biosynthesis and the expression of steroidogenic acute regulatory protein (StAR) which is crucial for the transport of cholesterol to mitochrondria where steroid biosynthesis is initiated [76], [77]. StAR transcription is considered to be the rate-limiting step in adrenal CORT biosynthesis [81].

Since the adrenal clock does not have direct access to environmental day/night signals, the adrenal clock is entrained to the light∶dark cycle by signals originating in the hypothalamic SCN clock that reach the adrenal via autonomic innervation from the splanchnic nerve [82], [83]. Therefore, in the absence of a light∶dark cycle (e.g., constant dark conditions), the plasma CORT rhythm free-runs in concert with other SCN-driven circadian rhythms such as wheel-running activity. Further evidence in support of the autonomic regulation of CORT secretion comes from studies showing a dissociation of ACTH and CORT in intact animals. In hamsters with behaviorally split circadian rhythms (two SCN-driven bouts of behavioral and physiological rhythms approximately 12 h out of phase per circadian cycle), CORT is rhythmically secreted in phase with each activity bout in the absence of any variation in plasma ACTH [84]. Moreover, stimulation of animals with light activates the splanchnic nerve and evokes CORT secretion in the absence of changes in plasma ACTH levels [26], [27]. Thus, the SCN clock, entrained to the day/night cycle via the retinohypothalamic tract, entrains the cellular clock mechanism of the adrenal via descending autonomic circuits, which in turn determine when the adrenal is most responsive to circulating ACTH.

The mechanism underlying the reduction in the daily CORT rhythm observed in highly phase delayed animals in the present study is not known. If altered entrainment to the light∶dark cycle had an effect on ACTH secretion, then a reduction in the amplitude of the daily ACTH rhythm might have contributed to the reduction in the daily CORT rhythm in highly phase delayed animals. The normal daily rise in plasma ACTH is correlated with the increase in adrenal responsiveness to ACTH and thus this low amplitude rhythm normally contributes to the pronounced daily increase in plasma CORT. Conversely, the lowered CORT levels observed in highly phase delayed animals might have resulted in reduced steroid negative feedback thereby producing increased secretion of ACTH and a higher amplitude ACTH rhythm. Plasma ACTH levels could not be determined in the current study due to the small volume of blood collected. Determining plasma ACTH levels in highly phase delayed animals will be useful for understanding the cellular mechanisms underlying the reduced CORT rhythm and will be addressed in future studies.

Light entrains the SCN clock via the RHT and the SCN entrains the adrenal through a multi-synaptic autonomic circuit. In animals with an adrenal cortex-specific clock gene knockdown, the plasma CORT rhythm is abolished in constant dark conditions. However, in these mice the plasma CORT rhythm persists when they are maintained in a light∶dark cycle suggesting that in the absence of an adrenal clock the autonomic innervation is sufficient to drive a CORT rhythm [77]. Since light can drive rhythmic CORT secretion independent of an effect of light on the SCN clock, retinal signals may have access to descending autonomic circuits via retinal fibers to hypothalamic sites outside the SCN that might entrain peripheral oscillators such as the adrenal [27], [85]–[88].

Light∶dark signals and SCN timing signals that are normally in phase may have become dissociated in highly phase delayed animals producing a mismatch between the rhythm in adrenal responsiveness to ACTH (light-driven) and the ACTH rhythm (SCN-driven) resulting in a reduction in peak CORT levels. The possibility that the adrenal clock remained entrained to the light∶dark cycle was examined. Although the population of animals available to test this hypothesis was small, the preliminary results suggest that the adrenal was not entrained to light offset in the highly phase delayed animals although the precise phase of the adrenal clock in highly phase delayed animals remains to be determined.

### Circadian phase delays in SAD

In a landmark clinical study, Lewy and colleagues reported that patients suffering from recurring depression during the short days of winter (seasonal affective disorder or SAD) had phase-delayed circadian rhythms relative to their sleep-wake cycle; morning light treatment phase advanced their circadian rhythms resulting in significant clinical improvement [89]. Although perhaps not the sole factor in the etiology of SAD, the altered circadian phase theory has gained considerable support since Lewy and co-workers' original observation [89] and the American Psychiatric Association reached a consensus in 2005 that light can serve as a first-line treatment intervention for seasonal affective disorder [90]. However, not all studies have reported circadian abnormalities in SAD patients and these conflicting results may be due to several factors [91]. One confounding factor is that group means may not represent individual circadian responses. Given the considerable variation noted in the phase angle of entrainment of C57BL/6J inbred WT mice under 8L:14D laboratory conditions in this current study (i.e., Ψs ranged from zero to >8 h after light offset) it might be considered remarkable that clinical studies of small groups of SAD patients have described significant circadian abnormalities. Indeed, when light treatment is timed to maximize the corrective phase advance for individual SAD patients a greater correlation is achieved between morning light treatment and clinical improvement [44], [92].

It is not known if the circadian system of individuals with SAD has a reduced sensitivity to light, although this has been suggested as one possible mechanism contributing to the illness [93]. Melanopsin expressing ipRGCs projecting to the SCN via the RHT mediate entrainment [5], [7], [8]. Recently a missense variant of the melanopsin gene has been described in SAD patients [94] and SAD patients exhibit lower retinal sensitivity to light [95]. It is likely that several factors contribute to the etiology of SAD, and among them may be a reduced sensitivity to light resulting from abnormalities in phototransduction in the retina [94], [95] or abnormalities in 5-HT neurotransmission in the SCN [17].

The diurnal rhythm of cortisol has been monitored in SAD patients with inconsistent results, although due to the suppressive effect sleep has on cortisol secretion [96], the endogenous cortisol rhythm may typically be masked in these clinical studies. Avery and colleagues conducted a well-designed and carefully controlled study with six SAD patients who were subjected to a 36 h constant routine paradigm that included 27 h of sleep deprivation to unmask the cortisol circadian rhythm. They reported that the diurnal cortisol rhythm was both phase delayed 2.4 h relative to body temperature nadir and attenuated, with the peak amplitude reduced ≈30% in the SAD patients relative to controls [46]. In the current study CORT rhythms were reduced ≈50% in animals with pronounced phase delays of >4 h.

Light can influence adrenocortical CORT secretion [26], [27] and thus another possible effect of altered entrainment of the activity rhythm to the L∶D cycle is that the plasma CORT rhythm might have remained phase-locked to the L∶D cycle rather than aligning with wheel-running behavior. Under this scenario the CORT rhythm would become out of phase with activity. Although blunted, the plasma CORT rhythm of animals with highly delayed Ψs appeared to remain in phase with the SCN-driven activity rhythm as evidenced by the diurnal rise in plasma CORT peaking around activity onset. No changes were evident in the phase of the CORT rhythm within the time points sampled in this study (i.e., −6, −3, −1, 1, 4, 7 h relative to activity onset) and thus we believe that more frequent sampling would not have detected a change in the phase of the CORT rhythm. In golden hamsters with the *tau* gene mutation that reduces the period of the SCN circadian oscillator by several hours, the daily rhythm in glucocorticoid secretion remains in phase with the SCN-driven activity rhythm [97]. It is highly unlikely that the diurnal rhythm of plasma CORT was significantly phase shifted relative to activity onset in phase-delayed animals in our study.

### Summary

In conclusion, significant modification of the normal pattern of entrainment of the SCN circadian timing system to the day/night cycle, discernible as a marked delay in the onset of the circadian rhythm of locomotor activity relative to light offset, has a profound effect on the diurnal rhythm of circulating CORT, reducing peak levels by 50%. The cellular mechanism underlying the dampened CORT rhythm is not known. It remains to be determined if a reduction in the amplitude of the daily CORT rhythm is associated with altered gene expression in the central nervous system and/or metabolic disruptions.

## Materials and Methods

### Ethics statement

The use of animals in this study was approved by the University of Nebraska-Lincoln Institutional Animal Care and Use Committee (ID 270) and the Colorado State University Institutional Animal Care and Use Committee (ID 96-298). All experiments were conducted in strict accordance with the recommendations in the Guide for Care and Use of Laboratory Animals of the National Institutes of Health and all efforts were made to minimize suffering.

### Animals

Adult (12–20 wk old) male wild type (n = 214) and 5-HT_1B_ KO (n = 106) mice were used. 5-HT_1B_ KO mice [98] on the C57BL/6 genetic background, originally provided by Dr. René Hen (Columbia University, New York, NY), were from our breeding colony. WT mice (C57BL/6J) were obtained from the Jackson Laboratory (Bar Harbor, ME).

### Activity rhythms

Animals were maintained individually in cages (24×14×13 cm) equipped with an activity wheel (11.5 cm diameter), with food and water constantly available, and housed in light-tight ventilated chambers (up to 6 cages to a chamber). Light in the chamber was provided by a 40-W fluorescent bulb masked at intervals such that each cage received similar illumination as measured at the floor of the cage (approximately 100 lux). The 5-HT_1B_ KO mouse breeding colony and all experimental animals were initially maintained in 12L:12D conditions (L = 100 lux; D = 0 lux). Computer-controlled light switches were used to generate the non-24 h light∶dark cycles.

Wheel-running activity was monitored as previously described [99]. Briefly, wheel revolution data were collected in 5-min bins, and activity records were generated in the standard manner, with each day's activity presented beneath that of the previous day. Data were analyzed using either CIRCADIA software (Dr. Ralph Mistlberger, Simon Fraser University) or ClockLab running within MATLAB (Coulbourn Instruments, Allentown, PA). The phase angle of entrainment of activity rhythms was defined as the time elapsed between lights off and wheel-running activity onset.

### Experimental protocols

#### Experiment 1

Animals (WT, n = 23; KO, n = 23) were group housed (3–4/cage) in a 12L:12D cycle for several weeks. Animals were then housed individually in cages equipped with an activity wheel. Locomotor activity was recorded for four days under 12L:12D conditions to confirm entrainment and for the remainder of the experiment after lighting conditions were changed to 9.5L:13.5D. After approximately 22–23 cycles in 9.5L13.5D, blood was collected one hour prior to activity onset determined for individual WT and KO mice. Blood was subsequently colleted at three other time points relative to activity onsets on cycle days 58, 64, 73 and 78 for plasma CORT determination.

#### Experiment 2

Animals (WT, n = 18; KO, n = 15) were maintained under 12L:12D conditions for several weeks and wheel-running activity was recorded for six days prior to blood collection. Blood was collected at three different time points relative to activity onsets of individual mice for plasma CORT determination.

#### Experiment 3

Wheel-running activity was recorded from 49 WT mice housed in 12L:12D for several weeks. Animals were arbitrarily divided into two groups; one group (n = 25) remained in 12L:12D and the other group (n = 24) was maintained in constant dark (DD) for 2–3 days. In this experiment trunk blood was collected from the animals at different time points relative to activity onsets, following halothane anesthesia and decapitation for subsequent plasma CORT determination.

#### Experiment 4

Wheel-running activity was recorded from WT (n = 52) and KO (n = 68) animals as in Experiment #1 in four independent experiments and blood was collected from 33 WT and 32 KO animals at one time point (one hour before activity onset) after 22–23 cycles in 9.5L:13.5D, the same number of cycles in 9.5L:13.5D after which blood was collected for that time point in Experiment 1. Blood was also collected at two additional time points in one experiment for plasma CORT determination.

#### Experiment 5

Wheel running activity was recorded from 36 WT mice maintained initially in 12L:12D for several weeks and then under 8L:14D conditions. After approximately 107 cycles, blood was collected from 19 animals one hour prior to activity onset for plasma CORT determination.

#### Experiment 6

Wheel-running activity was recorded from 36 WT mice maintained initially in 12L:12D for several weeks and then under 8L:14D conditions. Between cycles 71–78 adrenocortical responsiveness to ACTH was tested. Animals were challenged with exogenous ACTH (1–39) (Bachem Bioscience, King of Prussia, PA) after administration of 100 µg/kg (ip) dexamethasone sodium phosphate (Baxter, 10 mg/ml) [100]. Dexamethasone was injected 2 h prior to ACTH, which was injected 20 min prior to blood collection. All animals were injected with 0.1 ml vehicle or ACTH (0.2, 1.0, or 5.0 µg in 0.1 M phosphate buffer, pH 7.2 containing 0.3% bovine serum albumin [BSA]) at ZT11 and ZT 18. Plasma CORT values were not available for every animal as insufficient blood was collected during the 4 min allowed for blood collection. After ≈110 cycles in 8L:14D all animals were killed at either ZT12 or ZT18 and adrenal glands and brains were dissected for *in situ* hybridization histochemical analysis of clock genes.

### Blood collection and radioimmunoassays

The onset of wheel-running activity for individual mice was used as a marker of circadian phase to establish times for blood collection. Five minutes before the specified time of blood collection, animal rooms were entered; mice were removed from the light-tight chambers in their running-wheel cages and taken into an adjoining room where they were briefly anesthetized with halothane. Using a straight edge razor, ≈1 cm of the tail was clipped and a few drops of blood were collected and styptic powder was applied to stop the bleeding; animals were returned to their running-wheel cages and taken back to their light-tight chambers. In some experiments a maximum of 2 additional samples were collected on subsequent days by clipping 2–3 mm of tail [101]. Blood was immediately placed on ice and plasma was frozen until assayed for corticosterone, the only glucocorticoid found in mouse plasma [102]. The entire procedure from entering the room housing the chambers to putting the blood on ice took 3–4 minutes, thereby avoiding stress-induced increases in CORT. Importantly, chambers housing mice were opened for mouse removal only once per day. This precaution limited the number of animals that could be sampled in each experiment. When blood collection was scheduled for a time when animals were in the dark, infrared night vision goggles (ITT-NE5001, generation 3, GT Distributors, Austin, TX) were used for the procedure through anesthetizing the mice and then blood was collected under dim room light.

Corticosterone was measured by radioimmunoassay as previously described [103]. Briefly, plasma was diluted 1∶25 in 0.1 M phosphate buffered saline (PBS) (pH 7.0) and plasma-binding proteins that could interfere with antibody binding were heat denatured at 65°C for 1 h. Rabbit anti-corticosterone (MP Biomedicals, Orangeburg, NY) was used at a final dilution of 1∶2400. ^3^H-corticosterone (Perkin Elmer, Boston, MA; 70.0 Ci/mmol) was added to each assay tube at a concentration of 12,000 cpm/tube. Bound and free corticosterone were separated using dextran-coated charcoal. Standard curves consisted of dilutions of corticosterone (4-pregnen-11β, 21-diol-3,20-dione; Steraloids, Wilton, NH) ranging from 5–500 ng/ml. The sensitivity of this assay is 10 pg/tube. Inter-assay variation was 7.4% and intra-assay variation was 3.8%. All plasma samples were coded and were assayed in duplicate, blind to the time of collection and mouse genotype. Plasma CORT concentrations for Experiment 6 was determined via an enzyme-linked immunoassay kit (Assay Design, Ann Arbor, MI) according to manufacturer's specifications. The sensitivity of this assay is 1.3 ng/ml for a 2 µl sample, and the intraassay variability was 8.3% (all samples were run in the same assay to avoid interassay variability).

### Tissue collection, in situ hybridization histochemistry and image analysis

Brains and adrenal glands were quickly dissected. Brains were flash-frozen in a dry ice-isopentane bath at −30°C. Adrenal glands were submerged in a drop of M-1 embedding matrix and frozen on dry ice. Both brains and adrenal glands were stored at stored at −80°C until prepared for in situ hybridization histochemistry. Brain and adrenal tissue were sectioned using a Leica cryostat (model 1850, Bannockburn, IL) at −20°C to obtain 12 um coronal sections. Brains were sectioned through the extent of the SCN. Adrenal tissue was sectioned transversely through the middle of the gland at 12 um. Sections were thaw mounted onto poly-L-lysine-coated slides and stored at −80°C.

In situ hybridization for *per2* and *bmal1* mRNA was performed as previously described [53], [104]. Briefly, tissue was fixed in phosphate buffered 0.4% paraformaldehyde, washed in 2× standard sodium citrate (SSC), acetylated in 0.1M triethanolamine containing 0.25% acetic anhydride, washed in ultrapure water, dehydrated in ethanol, and air dried. S35-UTP labeled riboprobes were generated from plasmids containing a portion of the cDNA for mouse *per2* (nuclear transcript (nt) 2311–2939, Genebank accession no. NM_011066) and *bmal1* (nt 629–1210, Genebank accession no. NM_007489) as previously described [53]. The slides were incubated with hybridization buffer (50% formamide, 10% dextran sulfate, 2× SSC, 1× Denhardts solution, 0.1 mg/ml yeast tRNA in 50 mM sodium phosphate buffer, pH 7.4) containing the corresponding radiolabeled riboprobe overnight at 55°C. Following hybridization, slides were rinsed in 2× SSC, incubated in tris-buffered RNAase (200 mg/ml) at 37°C for 1 hr, washed in 0.1× SSC at 65C for 1 hr, rinsed in water, dehydrated in ethanol and air dried. Slides were exposed to film (Kodak BioMax) for 3 weeks and developed using standard methods. Autoradiograms were digitized using a lightbox (Northern Light B95), CCD camera (Sony XC-77), and Image J software (v. 1.41, NIH). Optical density was measured in the region of interest (SCN or adrenal cortex; 2–4 measurements per region, per animal) and subtracted from background (lateral hypothalamus, or adrenal medulla).

### Statistical analysis

Data are presented as means ± SEM. When experiments required comparison of two groups, statistical differences were determined by two-tailed Student's *t*-test. When multiple groups were compared, statistical differences were determined by one-way or two-way ANOVA. Specific differences were determined using Tukey's HSD post hoc analysis. Statistical significance was taken as P<0.05.
